# Urinary Amino Acid Alterations in 3-Year-Old Children with Neurodevelopmental Effects due to Perinatal Dioxin Exposure in Vietnam: A Nested Case-Control Study for Neurobiomarker Discovery

**DOI:** 10.1371/journal.pone.0116778

**Published:** 2015-01-13

**Authors:** Muneko Nishijo, Pham The Tai, Nguyen Thi Nguyet Anh, Tran Ngoc Nghi, Hideaki Nakagawa, Hoang Van Luong, Tran Hai Anh, Yuko Morikawa, Tomoo Waseda, Teruhiko Kido, Hisao Nishijo

**Affiliations:** 1 Department of Public Health, Kanazawa Medical University, 1-1 Daigaku, Uchinada, Ishikawa, 920-0293, Japan; 2 Biomedical and Pharmaceutical Research Center, Vietnam Military Medical University, Ha Noi, Vietnam; 3 School of Nursing, Kanazawa Medical University, 1-1 Daigaku, Uchinada, Ishikawa, 920-0293, Japan; 4 Department of Obstetrics and Gynecology, Kanazawa Medical University, 1-1 Daigaku, Uchinada, Ishikawa, 920-0293, Japan; 5 Faculty of Health Sciences, Institute of Medical, Pharmaceutical and Health Sciences, Kanazawa University, Kanazawa, Ishikawa, 920-0942, Japan; 6 System Emotional Science, Graduate School of Medicine and Pharmaceutical Science, University of Toyama, 2630 Sugitani, Toyama, 930-0194, Japan; Institute for Health & the Environment, UNITED STATES

## Abstract

In our previous study of 3-year-old children in a dioxin contamination hot spot in Vietnam, the high total dioxin toxic equivalent (TEQ-PCDDs/Fs)-exposed group during the perinatal period displayed lower Bayley III neurodevelopmental scores, whereas the high 2,3,7,8-tetrachlorodibenzo-p-dioxin (TCDD)-exposed group displayed increased autistic traits. In autistic children, urinary amino acid profiles have revealed metabolic alterations in the amino acids that serve as neurotransmitters in the developing brain. Therefore, our present study aimed to investigate the use of alterations in urinary amino acid excretion as biomarkers of dioxin exposure-induced neurodevelopmental deficits in highly exposed 3-year-old children in Vietnam. A nested case-control study of urinary analyses was performed for 26 children who were selected from 111 3-year-old children whose perinatal dioxin exposure levels and neurodevelopmental status were examined in follow-up surveys conducted in a dioxin contaminated hot spot. We compared urinary amino acid levels between the following 4 groups: (1) a high TEQ-PCDDs/Fs and high TCDD-exposed group; (2) a high TEQ-PCDDs/Fs but low TCDD-exposed group; (3) a low TEQ-PCDDs/Fs exposed and poorly developed group; and (4) a low TEQ-PCDDs/Fs exposed and well-developed group. Urinary levels of histidine and tryptophan were significantly decreased in the high TEQ-PCDDs/Fs and high TCDD group, as well as in the high TEQ-PCDDs/Fs but low TCDD group, compared with the low TEQ-PCDDs/Fs and well-developed group. However, the ratio of histidine to glycine was significantly lower only in the high TEQ-PCDDs/Fs and high TCDD group. Furthermore, urinary histidine levels and the ratio of histidine to glycine were significantly correlated with neurodevelopmental scores, particularly for language and fine motor skills. These results indicate that urinary histidine is specifically associated with dioxin exposure-induced neurodevelopmental deficits, suggesting that urinary histidine may be a useful marker of dioxin-induced neurodevelopmental deficits and that histaminergic neurotransmission may be an important pathological contributor to dioxin-mediated neurotoxicity.

## Introduction

We previously followed up on infant cohorts in dioxin contamination hot spots in Vietnam and reported considerable neurodevelopmental impacts of perinatal dioxin exposure including postnatal exposure by breast feeding, which were examined using the Bayley Scales of Infant and Toddler Development, Version 3 (Bayley III) when the infants were 4 months old [1–3]. Recently, we extended the follow-up period to 3 years of age in this infant cohort in Vietnamese hot spots of dioxin contamination and reported that increases in autistic traits were associated with perinatal 2,3,7,8-tetrachlorodibenzo-p-dioxin (TCDD) exposure [4].

Autism spectrum disorders (ASD) are neurodevelopmental disorders that are characterized by impairments in socioemotional interactions, communication, and repetitive or restricted behaviors. Typically, patients with autism are suspected to have a genetic predisposition. In a recent report, genomic sequencing of six children with autism revealed mutations in a gene that prevents several essential amino acids from being depleted, leading to the development of autism-related neurological problems [5]. Previous studies of the plasma and urinary amino acid profiles of patients with ASD have also suggested that metabolic alterations in amino acids that serve as neurotransmitters for excitatory and inhibitory neurons may be an important pathological aspect of ASD and that amino acid measurements can be a useful additional ASD diagnostic tool [6–8]. However, many children with ASD are picky eaters and refuse to consume certain foods [9], which is believed to contribute to the fact that children with ASD suffer from deficiencies in essential amino acids due to their poor protein intake.

Therefore, in the present study, we investigated possible differences in urinary amino acid levels in 3-year-old children with high perinatal dioxin exposure and lower neurodevelopmental scores compared to well-developed children with low exposure levels, which were estimated based on dioxin concentrations in the breast milk of mothers residing in the most highly polluted areas of Vietnam. In our analysis, we also performed dietary surveys and compared amino acid levels between the high—and low-exposure groups after adjusting for food intake.

## Methods

### Study population

The Thanh Khe district wards in Da Nang City, which are located within 3 km of Da Nang airbase, have been characterized as dioxin-contaminated areas due to the presence of Agent Orange and other herbicides that were stored and spilled there during the Vietnam War between 1961 and 1971 [10, 11] and were therefore selected as our study area. In collaboration with the Vietnamese government, Hatfield Consultants monitored environmental hazards in the area surrounding the Da Nang airbase, including the presence of heavy metals and organochlorines in the soil, and reported that dioxins were prominent among the hazardous chemicals found there [12].

Pregnant mothers were recruited for the present study by obstetricians upon arriving to give birth at the district hospital in 2008. The criteria for recruitment were as follows: (i) The mothers must have resided in one of the study areas for a period encompassing at least the duration of their pregnancy, and (ii) the mothers must have given birth to full-term babies without complications during childbirth. These mothers had no pregnancy-induced hypertension or pregnancy complication. A total of 159 mother-infant pairs were enrolled at baseline [1, 2]. Written informed consent was obtained from all mothers according to a process that was reviewed and approved by the Health Department of Da Nang City and the Vietnam Military Medical University. The institutional ethics board for epidemiological studies at Kanazawa Medical University approved the study design (License number: ES-74).

Follow-up examinations were conducted 4 months, 1 year and 3 years after birth to assess the development of the infants. In the 3 year follow-up survey, a total of 122 pairs participated, and 111 children underwent neurodevelopmental testing. Urinary analyses using a gas chromatograph equipped with a mass spectrometer (GC-MS) were performed for 26 children who were selected from children whose neurodevelopmental status had been examined; twelve poorly-developed (subscale score < 8 or composite scale score < 80 in any domain of Bayley III) children with higher TEQ-PCDDs/Fs levels in their breast milk (TEQ-PCDDs/Fs ≥ 20.0 pg-TEQ/g-lipid), including 6 children with higher TCDD levels and 6 children with lower TCDD levels, and 14 children with low dioxin exposure (TEQ-PCDDs/Fs < 11.5 pg-TEQ/g-lipid), including 10 well-developed children and 4 poorly-developed children, who served as controls. The TCDD cut-off value was 3.5 pg/g of lipids, which was reported as the threshold level for increased autistic traits and lower developmental scores in our previous study [4]. The ratio of boys to girls was 1:1 in all of the groups. Total ASRS scores which show autistic traits were 57.3 in high TEQ-PCDDs/Fs and high TCDD which was lower compared with well-developed children with 54.7 of total score, although the difference between two groups were not significant.

### Body size measurements, food surveys, and neurodevelopmental assessments using the Bayley Scales of Infant and Toddler Development, Ver. 3 (Bayley III)

Body size parameters for the infants, including weight and height, were measured at the survey site. The food intake of the children during the day prior to conducting the survey was obtained using a 24-hour recall method from the mothers or caretakers, and the estimated daily intake (g) of the main food groups, including milk and milk products, cereals, meat, fish, and eggs, was recorded. The Bayley III is a neurodevelopmental assessment measure that was used to assess the children’s development of cognition, language (including receptive and expressive communication), and motor skills, which were further divided into fine and gross motor skills. The children were tested by one examiner in the presence of each child’s caregiver at community health centers.

### Breast milk collection and quantification of PCDDs/PCDFs in breast milk

A breast milk sample was collected from each nursing mother 1 month after birth by hand expression with the assistance of a midwife or medical worker. Samples were placed in clean polyethylene containers and temporarily stored in freezers set at −4°C in the local health centers. The samples were frozen in dry ice for transport to Japan by airplane and were analyzed at the Center for High Technology, Kanazawa Medical University (Uchinada, Japan). Approximately 10 ml of breast milk from each sample was used to quantify the levels of 17 different 2,3,7,8-substitued polychlorodibenzo-p-dioxin (PCDD) and dibenzofuran (PCDF) congeners using high resolution GC-MS. The established method of analysis was previously described in detail [13]. The concentrations of the PCDD and PCDF congeners (PCDDs/Fs) were lipid-base calculated. Toxic equivalent factors for use in calculating the toxic equivalents (TEQ) of PCDDs/Fs (TEQ-PCDDs/Fs) were referenced from the WHO 2005-TEF [14]. The levels of TCDD and total dioxin in the breast milk of the mothers in the present study were 3- to 4-fold higher than those in the breast milk of mothers living in unsprayed areas [15]. In the four samples of them, dl-PCBs-TEQ was 12.8% (3.9 pg-TEQ/g lipid) of total TEQ, which is lower than that (38.1%; 2.1 pg-TEQ/g lipid) in 12 samples collected in northern Vietnam, suggesting that mainly PCDDs/Fs contribute to dioxin toxicity.

### Urinary sample collection and quantification of amino acids

Approximately 10 ml of spot urine was collected from each child after examination. The urine specimens were transported in cool containers and frozen at −4°C in the local health centers. The samples were transported to Kanazawa Medical University, Japan in containers with dry ice and kept frozen at −20°C prior to analysis. The urea in the urine was decomposed by pretreating the samples with 30 units of urease (Sigma, Alexander, USA) per 100 μL sample. Stable isotope-labeled compounds were used as internal standards for the quantification analysis to demonstrate the accuracy of the measurements. After the protein in each urine sample was precipitated, the residue was derivatized by adding 100 μL of N,O-bis-(trimethylsilyl) trifluoroacetamide (BSTFA) containing 1% trimethylchlorosilane (TMCS). The metabolites in the derivatized samples were analyzed using GC-MS with a 7890A gas chromatograph and a DB-5MS GC column (Agilent J&W, Santa Clara, CA, USA) coupled with a 5975A inert XL-mass selective detector (MSD; Agilent, Santa Clara, USA). The conditions used for GC-MS were as previously described [16]. The mass spectra data acquisition system was used in combination with GC-MSD Chem Station software (Agilent). The identified compounds, mass spectra and chromatographic retention times were compared with the reference databases of the National Institute of Standards (NIST) 2008 and Kanazawa Medical University. All urinary amino acids were corrected by the urinary Cr concentration, as determined using GC-MS. Since histamine and serotonin are synthesized from histidine and tryptophan, respectively, their levels in urine suggest alteration of histamine and serotonin in the brain. Other amino acids such as taurine in urine also indicate their levels in the brain.

### Data analyses

The SPSS (Statistical Package for the Social Sciences, Ver. 21.0) software package for Windows (IBM; Chicago, IL) was used for the statistical analyses of the data. The concentrations of dioxins in breast milk and urinary amino acids were base 10 logarithmically transformed to improve normality. A generalized linear model was used to compare the urinary amino acid levels in the four children groups at different dioxin levels after adjustment for covariates including maternal education (years). To analyze the relationships between neurodevelopmental scores and urinary amino acids, a linear regression model was performed with covariates, excluding the 4 poorly-developed children with low dioxin exposure. The results were considered to be significant if P < 0.05.

## Results

To investigate the relationships between urinary amino acids and dioxin exposure, differences in five urinary amino acid concentrations and their ratios to glycine (/G) at 3 years of age were investigated among the four groups of children: 1) The high TEQ-PCDDs/Fs and high TCDD-exposed children (high TEQ-high TCDD group), 2) the high TEQ-PCDDs/Fs and low TCDD-exposed children (high TEQ-low TCDD group), 3) the low TEQ-PCDDs/Fs-exposed and poorly-developed children (low TEQ/PD group), and 4) the low TEQ-PCDDs/Fs-exposed and well-developed children (low TEQ/WD group). Because the high TEQ-PCDDs/Fs-exposed children displayed lower communication scores on the Bayley III, we attempted to compare their urinary amino acid levels not only with the well-developed children who had been exposed to lower dioxin levels but also with the poorly-developed children who had been exposed to lower levels of dioxins.

### Characteristics of the four groups

Body sizes, levels of TCDD and TEQ-PCDDs/Fs, and Bayley III scores are shown in Table 1. The mean age with age-range at the survey was 38 with 34–39 months. Body size was the smallest in the high TEQ-high TCDD group, and the weights and heights of the children in this group were significantly lower than those in the low TEQ/WD group. However, birth weight and weight gain during 3 years in the high TEQ-high TCDD group were not significantly lower compared with low TEQ/WD group. In addition, no significant difference of head circumference at birth and 3 year old was found among these four groups.

Generally, Bayley III scores were lower in the high TEQ-high TCDD, high TEQ-low TCDD, and low TEQ/PD groups. However, all of the composite scores of cognitive, language, and motor functions were lower in the low TEQ/PD group, while the motor composite scores were not significantly lower in the two high TEQ-exposed groups. The language composite score was not significantly lower in the high TEQ-high TCDD group due to the wide standard deviation (Table 1).

**Table 1:** Mean body size, weight gain for 3 years, dioxin exposure levels, and neurodevelopmental scores in 3-year-old children exposed to different levels of dioxin exposure based on dioxin-TEQ and TCDD exposure.

	**Group I**		**Group II**		**Group III**		**Group IV**				
	**Low TEQ-WD (N = 10)**		**Low TEQ-PD (N = 4)**		**High TEQ-low TCDD (N = 6)**		**High TEQ-high TCDD (N = 6)**		**P-value**		
	**Mean**	**SD**	**Mean**	**SD**	**Mean**	**SD**	**Mean**	**SD**	**II vs I**	**III vs I**	**IV vs I**
Age (months)	37.2	1.6	37.3	0.2	36.2	1.8	37.2	1.2			
Gender (boys %)	50.0		50.0		50.0		67.0				
Gestational weeks	39.8	0.79	39.0	0.82	39.1	0.80	39.7	0.82			
Birth weight	3377	438	3113	179	3108	190	3120	505			
Head circum at birth	33.8	1.5	33.4	1.4	32.6	1.1	33.5	1.3			
Weight (kg) at 5 years old	13.8	1.0	12.9	1.6	12.3	0.8	11.8	1.6			0.041
Height (cm) at 5 years old	94.5	2.6	91.2	3.8	90.2	3.4	90.5	3.3			0.014
Head circum at 5 years	48.4	1.4	48.4	1.4	46.8	1.3	48.8	1.4			
Weight gain for 5 years	9.3	1.1	8.7	1.4	8.5	0.7	7.8	1.1			
***Dioxin in breast milk***											
TCDD (pg/g lipid)	0.9	1.5	1.3	1.3	2.5	1.1	5.2	1.4		0.001	0.001
Total TEQ (pg-TEQ/g lipid)	7.2	1.5	11.8	1.2	23.2	1.1	25.9	1.2		0.001	0.001
***Bayley scales***											
Cognitive	102.0	3.5	86.3	4.8	90.8	7.4	90.0	7.1	0.001	0.009	0.005
Language	100.3	6.1	79.3	4.5	92.7	3.4	81.5	14.7	0.001	0.035	
Receptive Communication	11.1	1.6	6.8	0.5	8.8	1.0	7.2	2.5	0.002		0.002
Expressive Communication	9.0	1.2	6.0	1.4	8.7	0.5	6.5	2.6			
Motor	107.3	6.7	88.8	3.8	98.7	5.9	94.5	19.3	0.001		
Fine motor	11.9	1.3	8.3	1.0	10.0	1.1	8.8	1.7	0.002		0.001
Gross motor	10.4	2.1	8.0	1.4	9.5	1.0	9.3	4.8			

Low TEQ: TEQ-PCDDs/Fs <11.5, High TEQ: TEQ-PCDDs/Fs ≥ 20 pg-TEQ /g lipid, low TCDD: <3.5 pg/g lipid, high TCDD ≥ 3.5 pg/g lipid, SD: standard deviation, WD: well developed, PD: poor developed, circum: circumference.

Comparisons were performed by one-way ANOVA with post hoc tests among groups by Sheffe or Dunnet t3.

### Urinary amino acid levels

Urinary concentrations of glycine, lysine, glutamine, histidine, tryptophan, and taurine following correction by creatinine were compared among the four groups of children (Table 2). The urinary histidine and tryptophan concentrations were significantly lower in the high TEQ-high TCDD group compared to the low TEQ/WD group. For the other urinary amino acids, including alanine, phenylalanine, leucine, isoleucine, valine, serine, tyrosine, threonine, and asparagine, no differences in their urinary concentrations were observed among the four groups (data not shown). Because urinary excretion of amino acids can be affected by age, gender, and body weight (muscle volume), urinary amino acid levels were compared among the four groups of children after adjusting for the covariates, including age, gender, and body weight. The estimated adjusted mean values of these four groups are shown in Table 2, revealing significantly decreased urinary histidine and tryptophan concentrations in the high TEQ-high TCDD and high TEQ-low TCDD groups. In particular, the adjusted mean concentrations of histidine and tryptophan in the high TEQ-high TCDD group were the lowest among the four groups. Additionally, the adjusted mean values of urinary histidine and tryptophan concentrations with an additional covariant, either milk intake or meat intake, were analyzed because food intake, particularly protein-rich food intake, is known to affect urinary amino acid levels. Even after adjusting for age, gender, body weight, and milk or meat intake, urinary histidine and tryptophan levels in the high TEQ-high TCDD group were the lowest among the four groups and were significantly lower than those in the low TEQ/WD. Similarly, urinary levels of histidine and tryptophan in high TEQ-low TCDD group significantly lower compared with low TEQ/WD group (Table 2).

**Table 2:** Comparison of urinary amino acid concentrations among children exposed to different levels of dioxin based on dioxin-TEQ and TCDD exposure after adjusting for age, gender, body weight, and food intake.

	**Group I**		**Group II**		**Group III**		**Group IV**				
	**Low TEQ-WD (N = 10)**		**Low TEQ-PD (N = 4)**		**High TEQ-low TCDD (N = 6)**		**High TEQ-high TCDD (N = 6)**		**P-value**		
	**Mean**	**SD**	**Mean**	**SD**	**Mean**	**SD**	**Mean**	**SD**	**II vs I**	**III vs I**	**IV vs I**
*no adjustment (mmol/mol creatinine)*											
Glycine	24.2	2.8	23.6	2.4	15.0	2.2	14.7	3.1			
Lysine	6.69	2.56	4.41	3.00	3.98	2.19	2.23	1.98			
Glutame	1.60	3.19	0.76	4.24	1.16	2.33	0.55	2.33			
Histidine	187.1	2.5	55.9	3.1	43.6	2.7	16.0	3.1			0.000
Tryptophan	2.70	2.31	1.53	1.76	1.03	2.17	0.64	1.53			0.002
Taurine	0.30	2.77	0.67	4.50	0.22	4.80	0.29	2.49			
*adjusting for age, gender, and body weight*											
Histidine	174.6	1.5	55.1	1.7	42.9	1.6	18.5	1.6		0.035	0.003
Tryptophan	2.68	1.30	1.55	1.45	0.95	1.41	0.70	1.40		0.033	0.009
*adjusting for age, gender, body weight, and milk intake*											
Histidine	175.8	1.5	62.7	1.8	46.9	1.6	16.9	1.7		0.059	0.004
Tryptophan	2.77	1.29	1.91	1.43	1.09	1.37	0.62	1.37		0.041	0.003
*adjusting for age, gender, body weight, and meat intake*											
Histidine	187.93	1.50	53.95	1.73	42.66	1.63	18.58	1.66		0.038	0.004
Tryptophan	2.99	1.32	1.54	1.45	1.00	1.42	0.69	1.41		0.030	0.007

Low TEQ: TEQ-PCDDs/Fs <11.5, High TEQ: TEQ-PCDDs/Fs ≥ 20 pg-TEQ /g lipid, low TCDD: <3.5 pg/g lipid, high TCDD ≥ 3.5 pg/g lipid, SD: standard deviation, WD: well developed, PD: poor developed.

Comparisons were performed after adjusting by age (months) at examination, gender, body weight at examination, and food intake by GLM analysis with least significant difference LSD test.

### Ratios of amino acids to glycine

To confirm the specific alteration of the urinary excretion of histidine and tryptophan, the ratios of five amino acids to glycine (lysine/G, glutamine/G, histidine/G, tryptophan/G, and taurine/G) were compared among the four groups of children (Table 3). Because urinary glycine levels were the second highest, they appeared to be the most stable among the four groups.

**Table 3:** Comparison of urinary amino acid ratios among children exposed to different levels of dioxin based on dioxin-TEQ and TCDD exposure after adjusting for age, gender, body weight, and food intake.

	**Group I**		**Group II**		**Group III**		**Group IV**				
	**Low TEQ-WD (N = 10)**		**Low TEQ-PD (N = 4)**		**High TEQ-low TCDD (N = 6)**		**High TEQ-high TCDD (N = 6)**		**P-value**		
	**Mean**	**SD**	**Mean**	**SD**	**Mean**	**SD**	**Mean**	**SD**	**II vs I**	**III vs I**	**IV vs I**
*no adjustment (amino acid / glysine)*											
Lysine/G	0.28	1.71	0.19	1.75	0.27	1.86	0.15	1.82			
Glutame/G	0.066	2.00	0.032	4.38	0.077	1.43	0.037	1.76			
Histidine/G	7.72	1.48	2.36	2.86	2.91	2.48	1.09	1.27			0.000
Tryptophan/G	0.112	2.05	0.065	2.74	0.069	1.79	0.044	2.22			
Taurine/G	0.012	2.41	0.028	2.56	0.014	5.28	0.020	4.25			
*adjusting for age, gender, and body weight*											
Histidine/G	7.08	1.26	2.30	1.39	3.18	1.33	1.17	0.01	1.352		0.000
*adjusting for age, gender, body weight, and milk intake*											
Histidine/G	7.50	1.22	1.70	1.33	2.73	1.26	1.37	1.28	0.001	0.006	0.000
*adjusting for age, gender, body weight, and meat intake*											
Histidine/G	6.64	1.29	2.31	1.41	3.16	1.36	1.16	1.37	0.025		0.001

Low TEQ: TEQ-PCDDs/Fs <11.5, High TEQ: TEQ-PCDDs/Fs ≥ 20 pg-TEQ /g lipid, low TCDD: <3.5 pg/g lipid, high TCDD ≥ 3.5 pg/g lipid, SD: standard deviation, G: urinary glycin e, WD: well developed, PD: poor developed

Comparisons were performed after adjusting by age (months) at examination, gender, body weight at examination, and food intake by GLM analysis with least significant difference (LSD) test.

Comparison of histidine/G was performed by one-way ANOVA with post hoc tests among groups by Dunnet t3.

Only the histidine/G ratio was significantly lower in the high TEQ-high TCDD group compared to the low TEQ/WD group. The ratios of histidine/G were also compared among the four groups after adjusting for age, gender and body weight. After adjusting for the covariates, the mean ratios of histidine/G in the high TEQ-high TCDD and high TEQ-low TCDD groups were significantly lower than those in the low TEQ/WD group (Table 3). Moreover, the histidine/G ratio was decreased in the high TEQ-high TCDD group compared to the low TEQ/WD group, even after adjusting for the three covariants and meat intake (Table 3).

### Neurodevelopmental functions and urinary amino acids

The multiple regression analysis (with adjustment for the covariates) between the urinary amino acids and the Bayley III scores of each function (cognitive, language, and motor) with subscales is illustrated in Table 4. Urinary histidine levels were significantly associated with the cognitive score, while the taurine/G ratio displayed a significant inverse association with the cognitive score; every 10-fold increase in histidine levels (P < 0.05) and every 10-fold decrease in the taurine/G ratio (P < 0.05) were associated with an 11.8 and 10.7 point increase in the cognitive score, respectively. Urinary histidine and tryptophan levels and the histidine/G ratio were significantly associated with the composite language and receptive communication scores, but histidine was only significantly associated with the expressive communication score. Specifically, a 10-fold increase in urinary histidine levels was associated with an 18.7 point increase (the greatest increase) in the composite language score (P < 0.01) and a 2.7 point increase in the receptive communication score (P < 0.05), suggesting that decreased urinary histidine levels were associated with lower language scores. All of the language scores (receptive and expressive communication and composite language) displayed inverse associations with the taurine/G ratio (P < 0.05). Urinary histidine and tryptophan levels and the histidine/G ratio were also associated with fine motor skills, with 2.1, 2.2, and 2.8 point increases in fine motor skill scores, respectively, suggesting that decreased urinary histidine and tryptophan levels and histidine/G ratios were associated with decreased fine motor scores. No urinary amino acid, with the exception of the taurine/G ratio, was associated with the gross motor skill score. Only urinary histidine levels were significantly associated with gross motor scores, with a 13.4 point increase for every 10-fold increase in urinary histidine.

**Table 4:** Bayley-III scores of 3-year-old children relation to adjusted^a^ to urinary amino acid levels.

	**Cognitive**	**Rec Com**	**Expres Com**	**Language**	**Fine motor**	**Gross motor**	**Motor**
*(mmol/mol creatinine)*							
Glycine	4.9	1.8	0.9	8.0	1.4	1.0	7.9
Lysine	4.5	1.7	1.2	8.4	2.1*	0.4	8.0
Glutamine	3.6	2.6*	0.6	9.1	1.6	0.8	7.8
Histidine	11.8*	2.7**	1.5*	18.7**	2.1**	0.7	13.4*
Tryptophan	6.3	3.1*	1.6	13.8*	2.2*	−0.2	6.5
Taurine	−5.7	−1.2	−1.2	−7.0	0.2	−1.4	−3.1
*(amino acid / glysine)*							
Lysine/G	−1.7	−0.6	0.6	0.0	1.6	−0.2	−0.6
Glutamine/G	2.8	2.7	−0.7	5.9	1.0	−0.2	2.5
Histidine/G	10.7*	3.5*	2.1	16.4*	2.8*	0.1	9.0
Tryptophan/G	0.7	1.3	0.7	6.1	0.7	−1.9	−3.6
Taurine/G	−8.5*	−2.3*	−1.7*	−11.7*	−0.7	−2.0*	−8.1

a: adjusted with covariates including age (days), gender, body weight at the survey, and meat intake, G: urinary glycine

*:P<0.05

**:P<0.01

## Discussion

### Specificity of histidine alteration

The present study demonstrated that perinatal dioxin exposure to TCDD and TEQ-PCDDs/Fs, as indicated by levels in breast milk, significantly decreased urinary excretion of histidine and tryptophan in 3-year-old Vietnamese children with lower neurodevelopmental scores in dioxin contamination hot spots. Urinary histidine and tryptophan were significantly decreased in children exposed to high levels of both total dioxin and TCDD with lower Bayley III scores. Moreover, children exposed to high levels of total dioxin but not TCDD, who displayed slightly lower neurodevelopmental scores on the Bayley III, also displayed decreased levels of histidine and tryptophan, although the impact was weaker in the children in this group than in the children who had been exposed to high levels of both TEQ-PCDDs/Fs and TCDD. In contrast, decreased histidine levels were not significant in children with poor neurodevelopment and low dioxin exposure compared to well-developed children exposed to low dioxin levels, suggesting that poor development itself is not a primary factor contributing to altered histidine metabolism. These results suggest that alterations of urinary histidine and tryptophan are specific to neurodevelopmental deficits resulting from dioxin exposure before and after birth when neurogenesis, especially myelination, continues [17].

### Histidine and brain function

In the present study, urinary levels of histidine were decreased in 3-year-old Vietnamese children who had been exposed to high dioxin levels. Furthermore, urinary histidine and the histidine/G ratio were associated with Bayley III scores in 3-year-old children, particularly with fine motor skill scores and not gross motor skill scores. These results suggest that low levels of urinary histidine are significantly associated with disturbances in the development of motor functions. Because histamine is synthesized from histidine, urinary depletion of histidine suggests histamine alteration in the brain. Consistent with this idea, TCDD administration induced histamine level alterations in the hypothalami of rats [18]. Histamine is an important modulator that acts as a trophic and/or neurogenic factor in the developing brain [19]. In the central nervous system, histamine is synthesized in the tuberomammillary nucleus in the hypothalamus, which sends histaminergic afferents to the cerebellum that are involved in motor coordination and precision, accurate timing of movements, and learning skills [20]. Therefore, dioxin exposure may affect histaminergic neurotransmission in the cerebellum, leading to poor development of fine motor skills.

In contrast, decreased urinary histidine levels in 3-year-old Vietnamese children who had been exposed to high dioxin levels may have been caused by alterations of histamine metabolism in the whole body. Lower urinary levels of histidine were observed in untreated patients with allergies whose histamine synthesis from histidine increased even as their plasma histidine levels were maintained at constant levels [21]. This finding suggests that the decreased urinary histidine levels in the dioxin-exposed children may be ascribed to increased histamine synthesis in the body, although no clear allergic symptoms, such as atopic skin and bronchial asthma, were observed in the present subjects. Exposure to dioxin-like compounds was reported to slightly increase allergic symptoms in an infant cohort study in Japan [22]. In children with ASD, urinary levels of essential amino acids, including histidine, were generally lower than those in control subjects [7, 23], and alterations in amino acid levels in the plasma have been reported [5, 6, 8], suggesting that changes in amino acid metabolism may contribute to the underlying pathology of ASD in children.

Recently, genetic variations in the histidine decarboxylase gene (the key enzyme that is responsible for the biosynthesis of histamine) that overlapped with those observed in ASD [24] were reported in patients with Tourette Syndrome, a developmental disorder characterized by the presence of both motor and vocal tics [25]. Castellan-Baldan et al. (2014) reported that histamine infusion into the brain reduced behavioral abnormalities, dopamine levels and D2/D3 dopamine receptor binding in the striata of histidine decarboxylase-knockout mice, suggesting that histidine decarboxylase deficiency in the basal ganglia alters histaminergic and dopaminergic neurotransmission, which may be an important pathological feature of Tourette Syndrome [26]. In schizophrenic patients who have similar sociobehavioral deficits to those in ASD, the levels of N-tele-methyl-histamine, a major brain metabolite of histamine, were increased in the cerebrospinal fluid, and H^1^ receptor binding sites were decreased [27]. Methamphetamine—and phencyclidine-induced animal models of schizophrenia also revealed increased histamine release in the brain [27]. These previous studies indicate that both increases and decreases in histamine in the brain may induce behavioral abnormalities. However, it remains unknown whether dioxin exposure enhances histamine synthesis from histidine or induces histamine depletion in the brain. Further animal studies are required to investigate histaminergic functions in the brain after exposure to dioxins. The present findings indicated that urinary histidine levels were significantly associated with neurodevelopmental deficits following dioxin exposure, which may be ascribed to alterations in the central histaminergic system.

### Tryptophan and brain function

Urinary tryptophan concentrations were also significantly decreased in the two groups of children who were exposed to high TEQ-PCDDs/Fs and were significantly associated with language and fine motor skill scores in the present study. Tryptophan is a precursor of serotonin that functions as a neurotransmitter and is related to the control of mood and emotional behavior. Several previous studies indicated that dysregulation of serotonin function may profoundly influence the severity of symptoms in certain individuals with autism [28, 29]. Croonenberghs et al. (2000) reported that plasma concentrations of tryptophan were significantly lower in a group of teenagers with autism than in age-matched controls, suggesting that the depletion of tryptophan is an important pathological feature of autism [30]. Recently, Boccuto et al. detected subnormal levels of the expression of certain genes that are involved in tryptophan metabolic pathways in patients with ASD [31]. In animals, TCDD exposure has been reported to increase serotonin levels in the brain and free tryptophan levels in the plasma, which are available for transport into the brains of the most TCDD-susceptible rats [32], suggesting that increased tryptophan in the brain increases serotonin synthesis and modifies TCDD exposure-induced anorexia. These findings also suggest that decreased urinary tryptophan levels may reflect tryptophan depletion in the body due to increased tryptophan levels in the brain and consequent increases in serotonin synthesis in children with lower neurodevelopmental scores and exposure to high levels of dioxin.

### Taurine and brain function

Although no significant differences in urinary taurine levels or the taurine/G ratio were detected among the groups that had been exposed to different levels of dioxin, inverse associations between the taurine/G ratio and neurodevelopment scores were observed for almost all functions (with the exception of fine motor skills) in the present study. These results suggest that the urinary taurine/G ratio, rather than taurine concentration, is a good marker of poor neurodevelopment, independent of dioxin exposure. Exogenous taurine has been reported to play a more important role in the developing brains of infants and children compared to the adult brain [33], and animal studies have indicated that taurine deficiency causes reductions in brain size and poor pyramidal cell and neuronal development [34, 35].

### Possible mechanisms of dioxin toxicity in brain development

It has been suggested that dioxin toxicity is mediated partly through aryl hydrocarbon receptor (AhR) [36], which up—and down-regulates expression of a large wide of genes related to growth and development, amino acid metabolism, and protein synthesis [37–39]. Consistently, dioxin exposure induced changes in various amino acid levels in the blood in animals [32, 40, 41], which might alter neurotransmitter metabolisms in the brain [32]. Furthermore, TCDD exposure has been reported to breach the blood-brain barrier, accumulate in the brain, and induce changes in neural gene and protein expression profiles in juvenile female rats in an AhR-dependent manner [42], although studies of AhR-mediated enzymatic alterations resulting from the conversion of amino acids to neurotransmitters in the brain have been limited. Therefore, the AhR may play an important role in dioxin toxicity by affecting the transport of amino acids and neurotransmitter synthesis in the brain. All of these alternations due to dioxin exposure could change brain and urine amino acid levels, and further suggest that urine amino acid levels could be used as biomarkers of dioxin exposure (see below). Further animal studies are required to investigate amino acid dynamics and the molecular mechanisms underlying the effects of dioxin in the brain.

### Clinical implications

Many studies have been performed to discover and develop biomarkers that contribute to the early and accurate diagnosis of neurodevelopmental disorders [7, 8, 30, 43–45], but reliable, non-invasive biomarkers that can be measured in small children have not yet been identified. Amino acid measurements in spot urine samples were examined in the present survey and can be easily and repeatedly observed in small children. Furthermore, urinary amino acid levels were significantly correlated with neurodevelopmental scores, and consequently, may be used as early biomarkers during mass screenings for neurodevelopmental deficits (termed neurobiomarkers) following dioxin exposure. However, the sample size in the present study was limited by the fact that all of the samples were drawn from subjects living in a single area. Therefore, to investigate the validity of urinary amino acid measurements in screening for neurodevelopmental deficits following dioxin exposure, we are now planning to analyze all of the children who were examined in the 3-year-old survey and reported to have autistic traits in our previous study [4]. Additionally, to determine whether these urinary amino acids can serve as reliable early markers of the effects of dioxin on neurological development, we are conducting a follow-up study in younger cohorts to clarify the associations between dioxin exposure and urinary amino acid levels prior to the appearance of neuronal symptoms.

### Limitations

The validity of the results and conclusions of the present study must be interpreted within the context of the study’s strengths and limitations. The neurodevelopmental test battery that was used (Bayley III) has some limitations with respect to the present study because this test was developed and standardized for infants and children in the United States rather than for Vietnamese infants; therefore, we could not judge the developmental levels of individual children based on the scores obtained using this test. However, the test was performed by a single well-trained examiner; thus, the associations between dioxin levels and test scores within this population should be reliable. Another limitation is that the neurodevelopmental status of those children with lower Bayley III scores was not clinically diagnosed.

### Conclusions

Urinary amino acid measurements were shown to be useful in detecting metabolic changes related to neurodevelopmental alterations in 3-year-old children who were perinatally exposed to dioxins. In particular, urinary histidine was suggested to be a candidate biomarker of neurodevelopmental deficits following dioxin exposure, and histaminergic neurotransmission may be an important pathological feature of dioxin-mediated neurotoxicity.

## Supporting Information

S1 File Raw Data File. (XLSX)
